# Do Management and Leadership Practices in the Context of Decentralisation Influence Performance of Community Health Fund? Evidence From Iramba and Iringa Districts in Tanzania

**DOI:** 10.15171/ijhpm.2016.130

**Published:** 2016-09-26

**Authors:** Chakupewa Joseph, Stephen Oswald Maluka

**Affiliations:** ^1^Mkwawa University College of Education, Iringa, Tanzania.; ^2^Institute of Development Studies, University of Dar es Salaam, Dar es Salaam, Tanzania.

**Keywords:** Community Health Fund (CHF), Leadership, Management, Tanzania

## Abstract

**Background:** In early 1990s, Tanzania like other African countries, adopted health sector reform (HSR). The most strongly held centralisation system that informed the nature of services provision including health was, thus, disintegrated giving rise to decentralisation system. It was within the realm of HSR process, user fees were introduced in the health sector. Along with user fees, various types of health insurances, including the Community Health Fund (CHF), were introduced. While the country’s level of enrolment in the CHF is low, there are marked variations among districts. This paper highlights the role of decentralised health management and leadership practices in the uptake of the CHF in Tanzania.

**Methods:** A comparative exploratory case study of high and low performing districts was carried out. In-depth interviews were conducted with the members of the Council Health Service Board (CHSB), Council Health Management Team (CHMT), Health Facility Committees (HFCs), in-charges of health facilities, healthcare providers, and Community Development Officers (CDOs). Minutes of the meetings of the committees and district annual health plans and district annual implementation reports were also used to verify and triangulate the data. Thematic analysis was adopted to analyse the collected data. We employed both inductive and deductive (mixed coding) to arrive to the themes.

**Results:** There were no differences in the level of education and experience of the district health managers in the two study districts. Almost all district health managers responsible for the management of the CHF had attended some training on management and leadership. However, there were variations in the personal initiatives of the top-district health leaders, particularly the district health managers, the council health services board and local government officials. Similarly, there were differences in the supervision mechanisms, and incentives available for the health providers, HFCs and board members in the two study districts.

**Conclusion:** This paper adds to the stock of knowledge on CHFs functioning in Tanzania. By comparing the best practices with the worst practices, the paper contributes valuable insights on how CHF can be scaled up and maintained. The study clearly indicates that the performance of the community-based health financing largely depends on the personal initiatives of the top-district health leaders, particularly the district health managers and local government officials. This implies that the regional health management team (RHMT) and the Ministry of Health and Social Welfare (MoHSW) should strengthen supportive supervision mechanisms to the district health managers and health facilities. More important, there is need for the MoHSW to provide opportunities for the well performing districts to share good practices to other districts in order to increase uptake of the community-based health insurance.

## Introduction

In early 1990s, Tanzania like other African countries, adopted health sector reform (HSR). The most strongly held centralisation system that informed the nature of services provision including health was, thus, disintegrated giving rise to decentralisation system.^1^ It was within the realm of HSR process, user fees were introduced to raise additional revenue for health systems. User fees are charges levied at the point of service use and are supposed to reduce frivolous consumption of health services, increase quality of services available and, in turn, increase utilisation of services.^2^ The introduction of user fees in the health sector reduced people’s access to health services, especially the poor and the most vulnerable groups of the society.^2-5^ To offset the negative effects of user fees, many low- and middle-income countries (LMICs) have introduced various types of health insurance schemes, including community-based health insurance (CBHI). CBHI schemes are noted for the principal role of a community’s involvement in raising, pooling, allocating, purchasing, and supervision of the health financing arrangement. Their beneficiaries are individuals with no form of financial protection or ability to cover the cost of healthcare services and the schemes are voluntary in nature.^6^ While CBHI schemes are criticised for the limited extent of resource generation and pooling, they have been shown to facilitate and improve access to healthcare services, especially among children and pregnant women^7,8^ as well as the rural poor and informal workers.^9^

Based on the potential benefits of the CBHI scheme, the government of Tanzania officially introduced Community Health Fund (CHF) through Act No 1 of 2001.^10^ According to the CHF Act, the objectives of the fund are to: mobilise financial resources from the community for provision of healthcare services to its members; provide quality and affordable healthcare services; and improve healthcare services management in the communities.^10^ The scheme started operating in 1996 as a pilot study in Igunga district. The CHF membership is given on voluntary basis in which a household contributes an annual payment. The annual payment for the household is defined by the respective districts. The amount paid by the household entitles a particular household to a basic package of health services at a primary healthcare facility and hospital level in some districts.^11^ The funds collected by the district are doubled by a “matching grant” from the central government.^12,13^ Poor households which are unable to pay are supposed to be issued CHF membership card or an exemption letter.^10^ The powers to issue exemptions is vested into the Ward Health Committee after receiving recommendations from the village council.^10^ At the district level, the CHF is managed by the Council Health Service Board (CHSB) with representatives from the district authorities, public healthcare providers, private healthcare providers, and the community. At the ward and village level, the Ward and Village Development Committees, respectively, and the Health Facility Committees (HFCs) are responsible for mobilising people to join the CHF, overseeing premium collections, evaluating CHF operations and granting exemptions to poor households which are unable to pay.^11^ The ministry responsible for health and the ministry responsible for local government are required to provide advice and technical support to the fund and monitor and evaluate the activities of the fund.^10^

Over the past decade, several systematic reviews that assessed the impact of the CBHI schemes on health status, the use of health services and financial protection have reported mixed results.^14-17^ In some case, these schemes have provided some form of reductions in out-of-pocket payments^14^ while in other cases there is no significant impact on out-of-pocket payments, the use of health services or health status.^17^

Likewise, in Tanzania studies that have assessed the performance of the CHF schemes have reported mixed results. In some districts in Tanzania, CHF has managed to provide financial protection against health shocks to their members by reducing the level of out-pocket payment for healthcare.^18^ Despite these achievements, the country level enrolment rate has remained as low as 9.2%.^19^

The widely cited factors for the low enrolment in CHF include low economic status of the households^20-22^; poor quality of health services in the health facilities, particularly shortage of drugs, lack of essential medical supplies, lack of diagnostic equipment, and long waiting hours among others.^23,24^

Although studies indicate that CHF in Tanzania is ineffective in its implementation, there are wide variations between districts.^11,25^ Some districts have recorded higher performance in terms of enrolment relatively above the national average, and other districts are still far below the national average. High performing districts are but not limited to Iramba (54%), Bariadi (40.9%), and Singida rural (27.2%). Districts with poor uptake of CHF in Tanzania include Liwale (8.0%), Rungwe (6.5%), and Iringa (10%). Other districts include Ulanga, Kyela, Lindi, and Mbinga.^25,26^

While studies that have assessed factors associated with uptake of CHBI exist, we are not aware of previous studies that have explored the role of leadership and management practices in the uptake of the CBHI schemes in LMICs. The recent systematic review reported that lack of funds, poor quality of healthcare, and lack of trust were the major reasons for low CBHI coverage in LMICs.^27^ This study, therefore, explored the role of management and leadership practices in the performance of CHF in the context of the decentralised health systems. Health managers, like other managers, perform four broad functions: planning, organising, leading, and controlling.^28^ While the debate about achieving universal health coverage in LMICs focuses more on financial constraints, there is increasing evidence that weak management and leadership capacity is a major obstacle to service delivery in many countries.^29,30^ This paper has the potential to advance knowledge on the role of leadership and management in the implementation of community-based health insurance schemes at this time when many LMICs are working towards progress to universal health coverage.

## Methods

### Study Settings

#### Tanzanian Primary Healthcare Structure and Governance

Tanzania operates a decentralised health system, organized around three functional levels: district (primary level), regional (secondary level), and referral hospitals (tertiary level). Within the framework of the ongoing local government reforms, regional and district councils have full responsibilities for delivering health services within their areas of jurisdiction, and report administratively to the Prime Minister’s Office Regional Administration and Local Government (PMO-RALG).

The district councils have mandate for planning, implementation, monitoring and evaluation of health services. Each council has a District Medical Officer (DMO) who heads the Council Health Management Team (CHMT) and is answerable to the District Executive Director (DED), the head of the council. CHMTs are responsible for provision of services in dispensaries, health centres and district or district-designated hospitals. The Regional Health Management Teams (RHMTs) are responsible for interpreting health policies at the regional level. The Ministry of Health and Social Welfare (MoHSW) is responsible for policy formulation, supervision, and regulation for all health services throughout the country, as well as playing a direct role in the management of tertiary health services.

The Ministry of Finance and Economic Affairs (MoFEA) manages the overall revenue, expenditure, and financing. Its duties include preparing the Central Government budget and determining expenditure allocations to different Government institutions. The President’s Office, Public Service Management (PO-PSM) assists in matters of personnel and administration pertaining to public service in the entire government system. This includes responsibilities for personnel policies, administration and coordination of training and recruitment.

#### Description of the Study Sites

The study was conducted in Iramba and Iringa rural districts in Singida and Iringa regions, respectively. Table 1 summarises key characteristics of the study sites.

**Table 1:** Key Characteristics of the Study Settings

	**Iramba District**	** Iringa District**
Population	236 282 people	254 032 people
Population growth rate	2.3%	1.6%
CHF enrolment rate	54.0%	10.0%
Hospitals	2	1
Health centres	6	6
Dispensaries	60	61
Divisions	7	6
Wards	31	25
Villages	143	143
Health workers available	43.0%	37.0%
Shortage of health workers	57.0%	63.0%

Abbreviation: CHF, Community Health Fund.

#### The Study Design

The study adopted an exploratory case study design focusing on two districts one being a high performing district and the other being a low performing district. The CHF performance in this study refers to increased enrolments, retention of members, provision of benefit package to members, and management of the CHF funds. The exploratory case study is appropriate in investigating phenomena characterized by a lack of detailed preliminary research, especially formulated hypotheses that can be tested. This study design provided researchers with a high degree of flexibility and independence with regard to the data collection. The “high” and “poor” performing district was based on the increased enrolments, retention of members, provision of benefit package to members, and management of the CHF funds. Iramba, the high performing district had CHF enrolment growing faster from 12% in 2009 to 54% in 2013. While Iringa district with poor performance had 1.7% of CHF enrolment in 2009 to 10% in 2013. In terms of provision of benefit packages to members, in Iringa district services were limited to one health facility where members had registered while in Iramba district, CHF members could access services at any primary healthcare facility within the district.

A sample of 78 respondents was used, 39 from each district. The sample was drawn from health providers, district health managers, and other health stakeholders in the two districts. The sampled respondents included members of the CHMT, CHSB, Health Facility Governing Committees (HFGCs), in-charge of the health facilities, the Community Development Officer (CDO), and district CHF coordinators. Purposive sampling technique was used to select respondents. The main criterion for the selection of the participants was their involvement in the management of the CHF. Table 2 summarises category of respondents in the two study districts.

**Table 2:** Distribution of Respondents (N = 78)

** Category of Population**	**Total**	
	**Iringa**	** Iramba**
CHMT	8	8
CHSB	4	4
Health providers at Health centre and dispensaries	7	7
Health centre committees	9	9
Dispensary committees	9	9
CHF coordinator	1	1
CDO	1	1
Total	39	39

Abbreviations: CHF, Community Health Fund; CHMT, Council Health Management Team; CHSB, Council Health Service Board; CDO, Community Development Officer.

In each district, 3 health centres and 4 dispensaries were purposively selected in consultation of the district health managers. The health facilities were selected mainly based on the geographical accessibility and operation of the CHF for at least 1 year. In Iringa district, health centres that were selected included Isimani, Kiponzelo, and Nzihi and dispensaries were Tanangozi, Kalenga, and Weru. In Iramba district, health centres that were involved in the research included Mgongo, Ndago, and Kyengege while dispensaries were Bomani, Mbelekese, and Misigiri.

#### Data Collection Techniques

Data were primarily collected by the first author using in-depth interviews involving key respondents. The purpose of in-depth interviews was to collect information from a wide range of people who had first-hand knowledge about the CHF operations. A semi-structured interview guide was developed to assist the interviews with key respondents. The use of semi-structured interview guide enabled participants to express their views and to elaborate on issues that they felt were most relevant and important. Interviews were carried out until saturation point was reached, meaning that no new concepts were emerging from the interviews.

#### Data Analysis

In-depth interviews as well as minutes of the committee meetings were analysed using thematic approach. The unit of analysis was individual respondents as well as teams. The data analysis involved a number of steps. The data analysis firstly involved a process of familiarisation with the data by listening to the audio-recordings and reading the transcripts and notes from the minutes several times and noting ideas. Secondly, the first author developed a code manual based on the research objectives of the study. A list of initial themes was drawn up, based on the objectives of the study. The process of theme generation was reviewed and refined by going back and forth between the themes and the codes, as well as between the themes and the transcripts until the final themes were defined. We, therefore, employed both inductive and deductive approaches (mixed coding) to arrive to the themes. Thirdly, the transcripts of each interview were read through and responses were identified and were then coded manually in accordance with the identified themes. Fourthly, data were sorted and grouped together under patterns that were considered accurate, complete, and generalisable. As patterns of meaning surfaced, similarities and differences were identified. Finally, data were summarised and synthesised, retaining as much as possible the key expressions of respondents. After this analysis, data were triangulated to allow comparison across different categories of respondents.

## Results

This section presents key findings of the study organised in five themes namely: characteristics of the district health managers; uptake of CHF in the study districts; personal initiatives of the district health managers to increase uptake of CHF; supportive mechanism exerted by district health managers; and motivation and incentive mechanisms.

### Characteristics of the District Health Managers

The age of the respondents ranged from 18 to 51. As indicated in Table 3, out of 16 district health managers, 75% were males and 25% were females. In terms of the level of education, 38% out of 16 managers were diploma holders and 62.0% were degree holders. There was no difference in the level of education for the district health managers in the two districts. Even in terms of gender, there was no marked difference in the levels of education for the district health managers.

**Table 3:** Demographic and Social Characteristics of the District Health Managers

	** Districts**									**Total**	
	**Iramba**					** Iringa**					
	**Gender**				**Gender**				** No.**	**%**
	**Male**	**Female**	**Total**		**Male**	**Female**	**Total**			
			** No.**	**%**			**No.**	** %**		
Age										
18-29	1	0	1	12.5	1	1	2	25.0	3	19.0
30-40	2	2	4	50.0	2	0	2	25.0	6	37.0
41-50	2	0	2	25.0	2	0	2	25.0	4	25.0
51-60	1	0	1	12.5	1	1	2	25.0	3	19.0
Total	6	2	8	100.0	6	2	8	100.0	16	100.0
Education										
Diploma	2	1	3	38.0	2	1	3	38.0	6	38.0
Degree	4	1	5	62.0	4	1	5	62.0	10	62.0
Total	6	2	8	100.0	6	2	8	100.0	16	100.0

In terms of experience of stakeholders in the health sector, the findings did not reveal any huge difference among the two districts. As indicated in Table 4, 43% out of 16 had been working in the health sector over five years, and 19% had worked in the health sector between 1-2 years.

**Table 4:** Experience in of the District Health Managers in the Health Sector

** Experience in the Health Sector **	** Districts **				**Total**	
	**Iramba**		** Iringa**	
	**No.**	**%**	**No.**	**%**	**No. **	**%**
1-2 years	2	25.0	1	12.5	3	19.0
2-3 years	1	12.5	2	25.0	3	19.0
3-5 years	1	12.5	2	25.0	3	19.0
5 years	4	50.0	3	37.5	7	43.0
Total	8	100.0	8	100.0	16	100.0

Source: Field Data, 2014.

However, interview with CHF coordinators in both districts revealed difference in experience. In Iringa district, the CHF coordinator had only 1 year of experience. In addition, apart from being the CHF coordinator, he had to attend other duties concerning community development which greatly affected his performance as a CHF coordinator.

*“Due to my little experience, I should have been left to concentrate on CHF matters only. You know, coming in the office without knowing what you will exactly deal with, dwarf my experience and above all jeopardize CHF performance in the district”* (IDI with CHF coordinator).

It was evident from the interviews that the duties of CHF Coordinator were too demanding and required enough time to handle them. These duties included preparing monthly, quarterly and annual reports and budget; coordinating all CHF-related activities in the district, ensuring proper filing and managing membership data, and provide technical support to the CHF stakeholders in the district. In Iramba district, the CHF coordinator had stayed in the position for 4 years and was seen to have greater command of the above stipulated job descriptions.

In terms of training and managerial skills, the findings indicate that the vast majority of the district health managers in Iramba district had attended some training on managerial skills. Likewise, in Iringa district, majority of the district health managers had attended training on managerial skills. The most cited managerial training courses included management and supportive supervision, health system management, human resources management, and health information management.

### Uptake of the Community Health Fund in Iramba and Iringa Districts

CHF was introduced in Iramba district in 1998. In that year, 5.5% of total households were CHF members. The registration declined from 5.5% to 0.1% between June 1998 and March 1999. Ten years later, that is in 2009, the CHF enrolment grew to reach 12.0%. Since then, the district has experienced drastic increase in the enrolment such that by 2013 the enrolment recorded 54% of CHF members.^25^ On the other hand, in Iringa district, CHF was launched in 1999 and in that year enrolment stood at 5%. Since then, there has been fluctuation in the CHF enrolment such that by 2009 enrolment drastically dropped to 1.7%.^31^ Table 5 summarises trend in CHF enrolment in recent years in the two study districts.

**Table 5:** CHF Enrolment Rates 2009-2013

**District**	** Year **	** No. of Household **	** Registered Household **	** Percentage of Enrolled Household **
Iramba	2013	43 756	23 756	54.0
	2012	41 822	13 752	38.0
	2011	39 756	8514	20.0
	2010	37 889	6200	14.0
	2009	36 001	5410	12.0
Iringa	2013	68 578	6230	10.0
	2012	63 200	4758	6.9
	2011	61 301	2681	3.9
	2010	58 789	1662	2.4
	2009	54 511	1230	1.7

Abbreviation: CHF, Community Health Fund. Source: District CHF reports 2013/2014.

### Personal Initiatives of the District Health Managers

The analysis of interviews revealed that in Iramba district there were a number of initiatives taken by the district health managers aimed at increasing the uptake of CHF compared to Iringa district. Such initiatives included monthly evaluation of CHF performance, formation of hypothetical households who join CHF, introduction of mobile CHF and referral system, overhauling CHF and user fee payment system as well as community sensitisation aimed at making people join CHF.

To start with, Iramba district conducted monthly review of CHF performance. This was done through monthly meetings involving HFCs. The MoHSW’s guideline requires that HFCs meet quarterly. In those meetings, the HFCs discussed achievements, challenges and prospects of CHF in their respective health facility and reports were submitted to the CHMT on a monthly basis. As a custom, CHMT met from first to fifth each month to discuss reports from each health facility. This was a pre-condition for the in-charge of a health facility to get a token, mostly transport allowance as well as per diem for one day. Health facilities whose reports were rejected were sternly warned and the in-charges of the health facilities were not given transport allowance and per diem. The vast majority of respondents reported that this initiative significantly increased enrolment of CHF in Iramba district. On the contrary, In Iringa district, respondents across all levels rarely reported initiatives taken to increase the uptake of CHF. It is not surprising that CHF enrolment in Iringa district was disappointingly low.

Secondly, in Iramba, the district health managers formed hypothetical households who were obliged to join CHF. Respondents reported that elders who were unable to pay CHF premiums were grouped in 10s which formed one household and the village government paid on their behalf. Also primary and secondary school students were grouped into 10s and 5s, respectively. Each primary school student paid TShs 1000 per year (equivalent to US$0.5), and those of secondary school paid TShs 2000 per year (equivalent to US$1) making total of TShs 10 000 of a single household. Those who were working in informal sector like petty traders (Machinga) and motorcycle drivers (Bodaboda) also formed hypothetical household of 10s and then joined CHF quite smoothly. The findings from all respondents revealed that this initiative helped significantly in increasing the uptake of CHF in Iramba district.

Thirdly, Iramba district’s attempt to introduce portable CHF and referral system were commended by respondents as an initiative that motivated members to join CHF. According to our respondents, in Iramba district, when CHF members got sick, they could access health services at any primary healthcare facility within the district. In terms of the referral system, CHF members were referred to the health centres and to the district hospital. It was found out that CHF members who were referred to the district hospital could utilize up to a maximum of TSh 20 000 (equivalent to US$10) from the CHF fund to cover expenses. The CHF members were required to pay additional costs, if any. In contrast, in Iringa district, the CHF benefits were limited to one primary healthcare facility (dispensary or health centre) closest to the residence of the member and were not transferable to other healthcare providers in the district.

Fourthly, Iramba district increased the rate of user fee payments in order to encourage households to enrol with CHF. The district council increased the amount of user fees from TShs 1000 to TShs 3000 at the dispensary level, and from TShs 1500 to TShs 4000 at the health centre. At the same time, the annual premium for the CHF was changed from TShs 5000 to TShs 10 000 per household. This initiative was taken by CHMT and approved by the Full Council in 2012. The vast majority of respondents reported that increase of the amounts of out-pocket payments significantly increased the enrolment to CHF. Commenting on the benefits accrued from overhauling payment scheme, one respondent remarked.

*“From the day the payment scheme was overhauled, it is very rare to find patients who pay at the point of service delivery. Almost all of them are CHF members. We are now used to the truth that those who pay at the point of service delivery are mostly new comers to our district”* (In-charge of the health facility).

In contrast, in Iringa district, the payment scheme for CHF and user fees were found out to have remained the same for over 15 years since the introduction of CHF in 1999. The annual premium for CHF was TSh 5000 while the use fee was TSh 2000 per facility visit. The analysis of interviews with the in-charges of the health facilities in Iringa district revealed that patients preferred to pay TShs 2000 to get treatment instead of paying TShs 5000 as CHF premium annually. According to the members of the HFCs, the vast majority of patients were not happy with the quality of health services provided in the primary healthcare facilities.

Furthermore, Iramba district had introduced an innovative and participatory sensitisation process. In Iramba, sensitisation was not only done by the members of CHMT, CHSB, or HFCs but also it encompassed all district council management team led by the District Commissioner (DC) and the DED. The analysis of interview with CHMT members revealed that CHF had been made a permanent agenda and so was widely discussed in formal and informal meetings. For example, every Friday heads of departments met with the DED to discuss various issues. The CHMT members reported that CHF was made a permanent agenda in those Friday meeting. The DC on his part had designed a slogan known in Kiswahili as “*Kuku mmoja CHF mwaka mzima kwa kaya*” meaning “selling one chicken enables a family to buy CHF premium for the whole year.” This slogan encouraged household members to keep chicken that would enable them to join CHF easily. Commenting on how sensitisation had re-energised CHF uptake one respondent put it this way:

*“Our success in CHF enrolment for three years now has not come by accident. Our sensitisation has penetrated the whole community. In Iramba, CHF is everywhere. Our hamlet chair persons, Village Executive Officers (VEOs), Ward Executive Officers (WEOs), Ward Councillors above all DCs and DEDs have done a commendable job”* (IDI with a CHSB member).

In contrast, in Iringa, the vast majority of respondents reported that sensitisation was rarely carried out. Although CHMT mentioned it as a way of recruiting new members and retaining the old ones, it was reported as a dormant exercise. This was made clear by the CHSB members who argued that they had not been involved in any sensitisation exercises for two years since they were chosen. Similarly, the majority of the in-charges of the health facilities who were interviewed eloquently reported that they had confined their sensitisation exercise to what may be termed as “indoor sensitisation.” That is, they largely relied on the sensitisations done by the health providers at the health facility.

### Supportive Mechanism Exerted by the District Health Managers

The study explored the extent to which health facility in-charges are provided with support for effective implementation of CHF. It was evident from the analysis of interviews that in Iramba respondents felt satisfied with the support provided by the district health managers. Supporting mechanism that were said to be exerted to the health facilities included but not limited to constant encouragement of HSF who work in remote areas, continued coaching of HSF, regular visits of the district health managers to the health facilities, frequent and friendly communication between district health managers and HSF. On the contrary, in Iringa district the vast majority of the CHMT members felt that the support provided by the district health managers to the health facilities was inadequate. One respondent exemplified it this way:

*“I don’t think we are delivering the needed support to our Health Facility Staff (HFSs). For instance, for a week now I have been seeing several in-charges coming to these premises asking for procedures of purchasing drugs since the drugs that had been sent by MSD was over, but no one attends them. You know, before being a CHMT member I was the in-charge of a health facility and I experienced the same. There are times you really lose hope”* (IDI with a CHMT member).

### Motivation and Incentive Mechanisms

The findings revealed that both Iramba and Iringa districts had a system of motivating their HSF and health committees. The marked difference was evidenced in the type of motivation, consistency and modality that were used in implementing motivation and incentive schemes.

### Motivation Mechanism to Health Facility Staff

The analysis of findings revealed that in Iramba, there was a clear schedule of providing motivation and incentive scheme to the HFSs contrary to Iringa. In Iramba district, it was evident that extra duty allowance was timely paid to HFS on a monthly basis based on their rank. Clinical officers received TShs 50 000, senior nurses received TShs 40 000 and auxiliary nurses were paid TShs 30 000. Additionally, on-call allowance was timely given on a monthly basis. Clinical officers were paid TShs 60 000 and nurses were paid TShs 30 000. When asked the extent to which they were satisfied with the incentives one respondent remarked this way:

*“I have worked in 5 different district councils to date. Sincerely, I have not seen the district council that pays extra duty allowance and on-call allowance timely like Iramba. I cannot keep this secret my brother; Iramba is exceptional when it comes to the question of motivating health providers* (IDI with in-charge of health facility).

It was revealed that the CHF was the main source of fund which was used to pay incentives to health providers and district health managers as well as health committees. Iramba district was able to generate enough funds from CHF. According to the 2013/2014, Comprehensive Council Health Plan (CCHP), Iramba district had collected TShs 227 690 495 from CHF in 2012/2013 and the district was expected to collect TShs 394 508 403 from CHF during 2013/2014 financial year. In Iringa district, on the contrary, findings revealed that even though extra duty and on-call allowances were stipulated in the guideline, the exercise was constrained by irregularity in paying the suggested amount. This problem was compounded by the councils’ heavy reliance on the basket fund (donor funds). It was evident that there was frequent delay in the disbursement of the basket fund to the district councils. This largely affected the payment of incentives to the health providers, district health managers as well as HFCs. For instance, in the financial year 2012/2013, Iringa district collected only TShs 34 269 100 from the CHF. One CHMT member in Iringa district recounted:

*“We have good plans that are, however, interfered by the government’s delay to disburse fund timely. Sometimes three months can elapse without receiving any coin from the government. How can we pay say on-call allowances under such circumstances? We are making all our efforts but we mostly prove failure”* (IDI with CHMT member).

### Motivation Mechanism to the Health Facility Governing Committees

The findings revealed that both Iramba and Iringa districts had a system of motivating their health committees. Like health providers and district health managers, the marked difference was evidenced in the type of motivation, consistency and modality that were used in implementing motivation and incentive schemes. The analysis of interviews revealed that in Iringa, HFC members were paid allowance of TShs 5000 after each meeting which were convened quarterly. Thus, annually the HFGCs received a total of TShs 20 000. However, the findings further revealed that HFGCs did not get the allowance immediately after the meeting. Sometimes, the payment was delayed until the next meeting which largely affected morale of the committee members.

In contrast, in Iramba district, payments for the HFGCs worked well. Committee members were paid after each meeting which was convened monthly. The chairperson and secretary of the committee were paid TShs 3000 and other committee members were paid TShs 2000. The committee members reported that they were paid immediately after the meeting. While the amount of allowance paid was reportedly small, the vast majority of the committee members felt satisfied and motivated. In addition, the fact that in Iramba the committees met monthly to discuss issues pertaining to the performance of their health facility and CHF in general, it largely increased the uptake of CHF. It needs to be noted that the MoHSW guideline stipulated HFGCs to meet quarterly. Additional meetings are held at the discretion of the respective district councils.

### Motivation and Incentives to the Council Health Service Board

In Iringa district, the CHSB members were paid a flat rate of TShs 80 000 after every meeting which were normally convened quarterly. The analysis of interviews revealed that in Iringa district, the CHSB members rarely got an opportunity to visit health facilities. They largely waited for reports from CHMT who normally did supportive supervision to the health facilities. One CHSB member complained:

*“I assure you, we have never visited any health facility. Believe me, this is my second tenure as a CHSB member. When I visit my nearest health facility for medical reasons, I am merely treated just like any other citizen because very few health providers know me as an CHSB member. 3-4 months can elapse without us engaging in any health-related matters until the meeting is convened. Under such a situation do you expect the CHF to perform better?”* (IDI with CHDB member).

In contrast, the CHSB in Iramba district displayed outwardly enjoyment of their position in the district. In Iramba district, the chairperson and the secretary of the CHSB were paid TShs 200 000 while other members of the board were paid TShs 150 000 after each meeting which were normally convened quarterly. Additionally, when the CHSB members visited health facilities they were paid allowances. One CHSB member noted:

“*We are so grateful to our district council for what they do to us, especially when we hear the cries that other CHSB face in other districts. The district has given us a wide room for us to perform our tasks without any interference. We are even privileged that when we attend medical care at Kiomboi hospital, we do not stand in queues.*”

## Discussion

This paper has described the experiences of the district and local level actors in implementing community health insurance in Tanzania. In this section, we expand on the key issues raised by the respondents with reference to other studies on the implementation of health policies in developing countries.

The findings of the study did not reveal major differences among district health managers in the two study districts in terms of demographic characteristics of managers, management education and training, and management practices, although in the overall Iramba (the high performing district) scored slightly higher than Iringa rural (the low performing district) on some indicators. There were substantial differences in favour of Iramba district on one key indicator of leadership namely: personal initiatives of the top-district level leaders and district health managers to get things done.

It is evident that the variation in the uptake of the CHF reported in this study was largely attributed to personal initiatives of the top-district level leaders and the district health managers. This finding corresponds with earlier studies on decentralised district health management in developing countries.^11,32^ For example, a study done in Indonesia found out no major differences between the study districts in terms of demographic characteristics of managers, management education and training, and management practices. However, there were important differences in two key leadership indicators namely: personal initiatives to get things done and fairness in handling staff disciplinary matters.^32^ Nevertheless, the authors felt that there was insufficient evidence to support the assumption that the differences in performance of district health system were related to the differences in the performance of district health managers.

However, qualitative studies done in Tanzania found out that managerial and leadership practices of the district health managers, including effective supervision and personal initiatives of the top-district health officials coupled with incentives, were the major factors for the good performance of the district health system.^11,33^ It was evident that in Iramba district the high rate of enrolment in CHF was largely attributed to the personal initiatives of the top-district leaders such as DED and the DC as well as the DMO. Such an initiative was seen in a number of ways like introduction of monthly evaluation of the performance for CHF, formation of hypothetical households, introduction of portable CHF health service package and referral system as well as overhauling of payment system. The CHF design in Iramba district which provided opportunity for the CHF members to access health services at any primary health facility in the district was more attractive. A recent study in Tanzania reported that people are usually mobile, which includes their occasional travelling from one place to another for various socio-economic or business commitments.^34^ This implies that the CHF design that requires members to access services in a fixed health facility which is closest to one’s place of domicile seems to be inappropriate.

The high performance of CHF in Iramba district was also due to effective support mechanism that was rendered to the health facilities by the district health managers. In Iringa, CHF was affected by absence of laudable attempt to support the HSF. In districts like Iringa where the HSF were modestly supported by the district health managers, they suffered from what Marberry^35^ calls job-related stress which is associated with low level of job satisfaction, high rate of burnout, absenteeism and notoriously high hesitancy to serve the community. Studies have indicated that subordinates who lack support from their managers rarely put extra effort, are distracted from job performance and less motivated to perform beyond maximum requirement.^36^ In Iramba district, on the other hand, as CHF uptake kept improving, HSF and managers continued to be creative, reflective, enthusiastic and ambitious in ensuring that CHF enrolment continued to increase. Bradley et al^37^ report that in districts where HSF receive the needed support from their superiors, the HSF experienced sense of self efficacy, felt motivated and satisfied and consequently performed impressively in discharging their duties.

Although human motivation has been considered by psychologists to be a very difficult undertaking,^38^ a body of evidence suggests that where motivation has been consistently applied, particularly to the HSF and health committees, has resulted into higher achievement of set standards even beyond.^39^ This has been true with regard to CHF performance in Iramba district than in Iringa district. Findings in Iramba revealed that HSF as well as health committees enjoyed reasonable motivation. This presumably had an impact in improving the uptake of CHF.

## Strengths and Limitations of the Study

This study relied primarily on the review of minutes and in-depth interviews with key actors involved in the management of the CHF in the study districts. The study focused on supply side factors that determine CHF performance (management and leadership from the supply side). We recognise that the demand factors such as household’s ability to pay may also influence the performance of the CHF. In addition, this study did not explore contextual factors in the two study districts which might also explain the observed differences in the performance of the CHF. Future studies could focus on both supply and demand side factors in order to understand better the actual practices of CHF scheme in Tanzania. Notwithstanding these limitations, the study provides good insights into factors for the diverse performance of the community-based health insurance scheme in Tanzania.

## Conclusion

This paper was meant to explore the role of decentralised district health management and leadership practices on the uptake of community health insurance in Tanzania. The paper adds to the stock of knowledge on CHFs functioning in Tanzania where the practice is still relatively new compared to other countries in Africa and elsewhere. By comparing the best practices with the worst practices, the paper contributes valuable insights on how CHF can be scaled up and maintained.

The findings of this study provide insufficient evidence to support the assumption that the differences in the performance of the study districts in terms of uptake of CHF is related to the differences in the leadership and management practices and behaviours of the district health managers. No major differences were found among district health managers in the two study districts in terms of management and leadership skills and behaviour, although in the overall Iramba district scored slightly higher than Iringa rural district on some indicators.

The variation in the implementation of the community health insurance scheme reported in this study suggests that district health managers and local leaders were able to exercise considerable discretion in the implementation of the CHF scheme. It is mainly due to the personal initiatives of the top-district health leaders, particularly the district health managers and local government officials which led to the variations in the performance of the two study districts in terms on the uptake of the CHF. Additionally, availability of health services, effective supervision mechanisms, and incentives for the HFCs and board members had significant impact on the performance of the districts. The policy-makers, particularly the MoHSW and RHMTs, should strengthen supportive supervision mechanisms to the district health managers and health facilities. More important, there is need for the MoHSW to provide opportunities for the well performing districts to share good practices to other districts in order to increase uptake of the community-based health insurance. Furthermore, there is need for active collaboration between the district health managers and local level leaders such as councillors, ward and village leaders, as well as HFCs. These categories of actors are very important in the implementation of the policies.

## Acknowledgments

This work was part of the dissertation of the first author for Master of Arts in Development Management submitted at the University of Dar es Salaam, Dar es Salaam, Tanzania. The study was supported by SIDA SAREC. We are also grateful to the local government officials, district health authorities, and other stakeholders in the study districts for participating in the study. The views expressed in this study are interpretation of the authors of data received in the study areas. No blame should be laid on the supporting individuals and/or institutions.

## Ethical issues

The research on whose data this paper is based was approved by the University of Dar es Salaam, Dar es Salaam, Tanzania on behalf of the Government of Tanzania and the Tanzania Commission for Science and Technology. The research clearance was also approved by the regional and district authorities in their respective administrative areas. Verbal informed consent was sought from prospective respondents before starting the interview. All respondents were granted rights to withdraw from the interview any time they wished. Confidentiality of information given by informants was rightly observed. All interviews were tape recorded with participants’ consent and transcripts were stored in a manner that protected confidentiality.

## Competing interests

Authors declare that they have no competing interests.

## Authors’ contributions

CJ conceived the study, participated in data collection and analysis and drafted the manuscript. SOM supervised data collection and analysis and provided major contributions to the manuscript. Both authors read and approved the final manuscript.

## Authors’ affiliations

^1^Mkwawa University College of Education, Iringa, Tanzania. ^2^Institute of Development Studies, University of Dar es Salaam, Dar es Salaam, Tanzania.

## Key messages

**Implications for policy makers**

- The regional health management team (RHMT) and the Ministry of Health and Social Welfare (MoHSW) should strengthen supportive supervision mechanisms to the district health managers.
- The MoHSW should provide opportunities for the well performing districts to share good practices to other districts in order to increase uptake of the community-based health insurance.
- The MoHSW and the Ministry responsible for regional administration and local government need to make sure that incentives to the health providers, Health Facility Committees (HFCs) and board members are available and paid in time.

**Implications for public**

In order to effectively implement community-based health insurance, there is need to actively engage all stakeholders, including the public in the implementation of policies at the local level. The public, through health facility (user) committees should actively participate in monitoring the availability of quality health services in the health facilities.
